# O Volume do Apêndice Atrial Esquerdo Prediz a Recorrência de Fibrilação Atrial após Ablação por Cateter de Radiofrequência: Uma Metanálise

**DOI:** 10.36660/abc.20220471

**Published:** 2023-03-07

**Authors:** Zhenghao Liu, Xiaofei Mei, Hezi Jiang, Yujie Cui, Weiwei Yin, Kuangyi Wang, Tan Chen, Yafeng Zhou

**Affiliations:** 1 Medical Center Soochow University Suzhou Dushu Lake Hospital Suzhou China Department of Cardiology, Dushu Lake Hospital Affiliated to Soochow University, Medical Center of Soochow University , Suzhou Dushu Lake Hospital , Suzhou – China; 2 Institution for Hypertension Soochow University Suzhou China Institution for Hypertension of Soochow University , Suzhou – China

**Keywords:** Fibrilação Atrial, Ablação por Radiofrequência, Apêndice Atrial, Metanálise

## Abstract

**Fundamento:**

A influência do volume do apêndice atrial esquerdo (VAAE) na recorrência de fibrilação atrial (FA) após ablação por cateter de radiofrequência permanece obscura.

**Objetivos:**

Realizamos uma metanálise para avaliar se o VAAE é um preditor independente de recorrência de FA após ablação por cateter de radiofrequência.

**Métodos:**

Os bancos de dados PubMed e Cochrane Library foram pesquisados até março de 2022 para identificar publicações avaliando o VAAE em associação com a recorrência de FA após ablação por cateter por radiofrequência. Foram encontrados 7 estudos que preencheram os critérios especificados de nossa análise. Usamos a Escala de Newcastle-Ottawa para avaliar a qualidade dos estudos. Os efeitos agrupados foram avaliados dependendo das diferenças médias padronizadas (DMPs) ou *hazard ratios* (HRs) com intervalos de confiança (ICs) de 95%. Valores de p < 0,05 foram considerados estatisticamente significativos.

**Resultados:**

Um total de 1.017 pacientes de 7 estudos de coorte com um seguimento médio de 16,3 meses foram incluídos na metanálise. Dados de 6 estudos (943 indivíduos) comparando VAAE mostraram que o VAAE basal foi significativamente maior em pacientes com recorrência de FA em comparação com aqueles sem FA (DMP: −0,63; IC de 95%: −0,89 a −0,37; todos os valores de p < 0,05; I ^2^ = 62,6%). Além disso, maior VAAE foi independentemente associado a um risco significativamente maior de recorrência de FA após ablação por cateter de radiofrequência (HR: 1,10; IC de 95%: 1,02 a 1,18).

**Conclusões:**

A metanálise mostrou que existe uma correlação significativa entre o VAAE e a recorrência de FA após ablação por cateter de radiofrequência, e o papel do VAAE em pacientes com FA não deve ser ignorado na prática clínica.

## Introdução

A fibrilação atrial (FA) é a arritmia cardíaca mais comum, com prevalência mundial de cerca de 46,3 milhões de indivíduos em 2016. ^1^ A FA pode levar a acidente vascular cerebral, insuficiência cardíaca, demência e até à morte, com alto índice de incapacidade e fatalidade, assim causando enormes prejuízos médicos e encargos socioeconômicos em todo o mundo. ^2^ A ablação por cateter é mais benéfica do que a terapia médica convencional na restauração do ritmo sinusal e na qualidade de vida a longo prazo em pacientes com FA. ^3^ O isolamento da veia pulmonar continua sendo a pedra angular do tratamento por cateter para FA paroxística e persistente. No entanto, dependendo da estratégia de ablação e do tipo de FA, as taxas de sucesso do isolamento das veias pulmonares após 1 ano variam consideravelmente, de 50% a 80%. ^4^ A taxa de sucesso global de 1 ano da ablação de FA, aplicando a definição de sucesso fornecido no documento de consenso de 2017 (ausência de até mesmo um único episódio de 30 segundos ou mais de FA/taquicardia atrial/flutter atrial após o período de *blanking* de 3 meses sem medicamentos antiarrítmicos), foi observada em aproximadamente 52%. Existem vários preditores de recorrência de FA após ablação por cateter na literatura, tais como: idade avançada; sexo feminino; tipo de FA; predisposição genética; comorbidades coexistentes, incluindo obesidade, apneia do sono, síndrome metabólica, hipertensão, insuficiência cardíaca e valvopatia cardíaca; e grau de dilatação e cicatrização do átrio esquerdo. ^5^

A importância de estudar o apêndice atrial esquerdo (AAE) vem crescendo exponencialmente, visto que tem desempenhado papel vital na FA. Existe uma relação estreita entre o nível de velocidade de fluxo do AAE e a frequência de trombos e contraste de eco espontâneo como parâmetros qualitativos de risco tromboembólico elevado. ^6^ Além disso, de acordo com o ensaio BELIEF, o isolamento do AAE fora das veias pulmonares melhorou o prognóstico em pacientes com FA persistente de longa data. ^7^ Estudos recentes confirmaram que o volume do apêndice atrial esquerdo (VAAE) está envolvido na recorrência de FA. ^8 - 14^ No entanto, esses resultados são conflitantes com alguns artigos relatando que o VAAE tem uma correlação fraca ou mesmo irrelevante em pacientes com FA. ^15 , 16^

Portanto, resultados de estudos anteriores não foram resumidos quantitativamente em uma metanálise. Realizamos uma metanálise desses estudos para esclarecer se o VAAE basal foi preditivo para a recorrência de FA após a ablação por cateter.

## Métodos

Realizamos esta metanálise de acordo com as diretrizes MOOSE (Metanálise de Estudos Observacionais em Epidemiologia) ^17^ e PRISMA (Itens de Relatório Preferidos para Revisões Sistemáticas e Metanálises). ^18^ Visto que nossa metanálise foi baseada em estudos publicados anteriormente, não foram necessários a aprovação ética e o consentimento dos pacientes.

### Estratégias de pesquisa

Realizamos buscas nas bases de dados PubMed e Embase usando os termos seguintes: “left atrial appendage”, ”ablation”, ”recurrence” e “atrial fibrillation” (“apêndice atrial esquerdo”, “ablação”, “recorrência” e “fibrilação atrial”). A busca foi limitada a estudos em humanos publicados em inglês. Também pesquisamos manualmente as listas de referências dos artigos originais e de revisão relacionados para possíveis estudos. A pesquisa final da literatura foi realizada em 1º de março de 2022.

### Seleção dos estudos

O objetivo do nosso estudo foi avaliar a associação entre o VAAE basal e a recorrência de FA após ablação por cateter. Portanto, incluímos relatórios retrospectivos de qualquer um dos seguintes resultados: (1) diferenças médias de VAAE entre pacientes com ou sem recorrência de FA após ablação por cateter ou (2) riscos relativos multivariáveis ajustados de recorrência de FA após ablação por cateter com base no aumento por unidade do VAAE basal. Foi exigido para todos os estudos um seguimento mínimo de 6 meses após a ablação por cateter. Para esses estudos, o VAAE foi avaliado por uma ou várias das seguintes modalidades: ecocardiografia transtorácica, ecocardiografia transesofágica, tomografia computadorizada cardíaca ou ressonância magnética.

### Extração de dados e avaliação de qualidade

Dois autores (L e M) realizaram independentemente a busca da literatura, a extração de dados e a avaliação da qualidade de acordo com os critérios de inclusão predefinidos. As discrepâncias foram resolvidas por consenso. Os dados extraídos incluíram as características do paciente, número de pacientes com FA incluídos, estudos observacionais retrospectivos ou prospectivos, idade média, sexo, tipo de FA e proporções de pacientes com doença arterial coronariana, detalhes dos procedimentos de ablação por cateter, duração do seguimento e estratégias para detectar a recorrência de FA. Para os dados do resultado, incluímos estudos que preencheram todos os critérios anteriores e incluíram as diferenças médias padronizadas (DMPs) do VAAE basal em pacientes com e sem recorrência de FA com *hazard ratio* (HR) e intervalo de confiança (IC) de 95% do VAAE como preditores de recorrência de FA. A qualidade dos estudos incluídos foi avaliada pela Escala de Newcastle-Ottawa, ^19^ que julga a qualidade de cada estudo de coorte em relação aos 3 aspectos seguintes: seleção dos grupos de estudo, comparabilidade dos grupos e determinação do desfecho de interesse.

### Análise estatística

Para a análise da média do VAAE em pacientes com recorrência de FA, os valores médios do VAAE foram extraídos para pacientes com recorrência de FA e pacientes sem recorrência de FA, e foram calculados DMPs e ICs de 95% para cada estudo. Para analisar o risco de recorrência de FA após ablação por cateter de radiofrequência com base no VAAE, usamos HRs padronizadas com ICs de 95% para avaliar as diferenças no VAAE entre pacientes com ou sem recorrência de FA para as metanálises. Para estudos que relataram apenas *odds ratio* (OR), os valores de OR usando o modelo de riscos proporcionais de Cox univariado e multivariado em cada estudo primário foram diretamente considerados como HRs. Valores de p < 0,05 foram considerados estatisticamente significativos. Foram realizados o teste Q de Cochran e o teste I ^2^ para avaliar a heterogeneidade entre os estudos. I ^2^ > 50% indicou heterogeneidade significativa. Foi usado um modelo de efeito aleatório ou de efeito fixo dependendo da heterogeneidade calculada. Para o VAAE médio na análise da recorrência de FA, a estatística Q (p = 0,013) e o índice I ^2^ de 62,6 indicaram heterogeneidade significativa. Para análise dos estudos que relataram o risco de recorrência de FA com base no VAAE, estatística Q (p = 0,00) e índice I ^2^ de 90,2 indicaram heterogeneidade grave, novamente levando-nos a adotar o modelo de efeito aleatório para agrupar tamanhos de efeito. Um modelo de efeito aleatório foi aplicado para sintetizar os resultados, porque este é um método mais generalizado que incorpora a heterogeneidade dos estudos incluídos ao combinar os resultados. Foram realizadas análises de sensibilidade, removendo estudos individuais um de cada vez, para avaliar a estabilidade dos resultados. ^20^ Gráficos de funil e testes de regressão de Egger foram realizados para avaliar o possível viés de publicação. ^21^ Todos os testes estatísticos foram realizados com STATA, versão 15.0 (StataCrop, College Station, TX, EUA).

## Resultados

### Resultados da pesquisa da literatura

**Figura 1:**
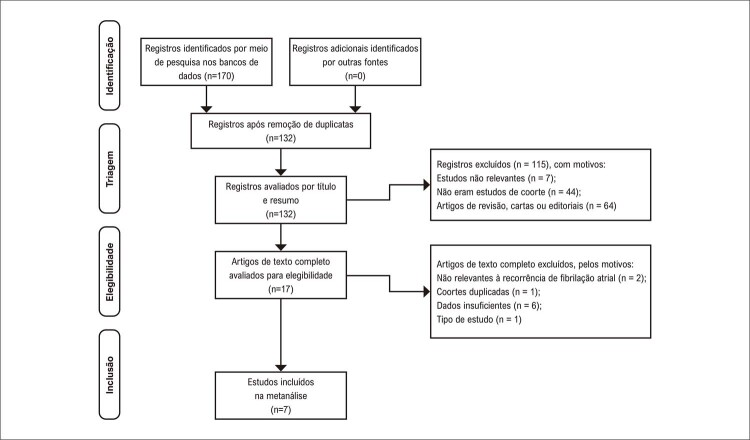
– Fluxograma de identificação da literatura.

A Figura 1 mostra o processo de pesquisa e estudo do banco de dados. Resumidamente, 170 estudos foram obtidos por meio de nossa pesquisa inicial na literatura. Após a remoção de duplicatas, 132 artigos foram triados por título e resumo, e 115 deles foram excluídos (7 estudos não eram relevantes; 44 não eram estudos de coorte; 64 eram artigos de revisão, cartas ou editoriais). Os 17 estudos restantes foram submetidos à revisão de texto completo. Destes, 10 estudos foram excluídos pelos seguintes motivos: não relevante à recorrência de FA (n = 2), dados insuficientes (n = 6), tipo de estudo (n = 1) e coortes duplicadas dos estudos incluídos (n = 1). Por fim, 7 estudos preencheram os critérios e foram incluídos em nossa análise.

### Características do estudo e avaliação de qualidade

As características dos estudos incluídos estão listadas na Tabela 1 . Em total, nossa metanálise incluiu 7 estudos de coorte retrospectivos com um total de 1.017 pacientes com FA submetidos à ablação por cateter. A duração média de seguimento dos estudos foi de 16,3 meses. Um estudo incluiu exclusivamente pacientes com FA paroxística, ^11^ e um outro incluiu apenas pacientes com FA persistente, ^12^ enquanto os outros incluíram ambos os subtipos de FA. Alguns estudos avaliam o VAAE usando um ou vários métodos, incluindo: ecocardiografia transtorácica, ecocardiografia transesofágica, tomografia computadorizada e ressonância magnética. Seis dos estudos incluídos realizaram apenas isolamento das veias pulmonares, enquanto um estudo realizou ablação linear adicional durante a ablação de FA. Os estudos incluídos eram geralmente de boa qualidade, com a Escala de Newcastle-Ottawa variando entre 6 e 9.

**Tabela 1:** – Características do estudo

Estudo, ano	Região	Número de pacientes	Desenho do estudo	Sexo masculino, %	Idade, anos	Tipo de FA (paroxística, %)	Tempo de seguimento, meses	Hipertensão, %	Diabetes, %	DAC, %	VAAE médio		Tipo de imagem usado	Detalhes da ACF	Período de blanking, meses	Avaliação para detectar recorrência	NOS
											Recorrência	Sem recorrência					
Simon J, 2022. ^14^	Europa	561	Estudo retrospectivo unicêntrico	65,1	61,9 ± 10,2	40,8	NR	73,3	14,6	9,1	8,8 ± 5,2	7,6 ± 3,2	TCC	ICVP	3	ECG ou ECG Holter	9
Du W, 2020. ^8^	Ásia	108	Estudo retrospectivo unicêntrico	53,7	63,1 ± 8,1	65,7	12	NR	17,6	18,5	13,34	9,67	TCC, ETE, ETT	ICVP plus	3	ECG ou ECG Holter	7
Tian X, 2020. ^9^	Ásia	83	Estudo retrospectivo unicêntrico	59	60,4 ± 10,1	65,7	19	NR	NR	NR	8,53	15,8	TCC	ICVP	3	Holter	9
Teixeira P, 2017. ^10^	Europa	52	Estudo retrospectivo unicêntrico	58	54,4 ± 9,7	57,7	24	NR	NR	NR	11,3	8,2	TCC	ICVP	3	ECG ou ECG Holter	7
Gul E, 2017. ^11^	América do Norte	59	Estudo retrospectivo unicêntrico	44	64,6 ± 9,8	0	13	69	20	19	11	9,7	TCMD, ETT	ICVP	3	Holter	8
He Y, 2018. ^12^	Ásia	80	Estudo retrospectivo unicêntrico	60	57,3 ± 10,4	100	12	NR	NR	NR	13,3	11,2	ETE, ETT	ICVP	3	ECG ou ECG Holter	8
Suksaranjit P, 2018. ^13^	América do Norte	74	Estudo retrospectivo unicêntrico	68	72 ± 11	40	18	65	7	11	NR	NR	RM-RTG	ICVP	Holter	3	7

*Todos os estudos adotaram nível de significância de p < 0,05. ACR: ablação por cateter de radiofrequência; DAC: doença arterial coronariana; ECG: eletrocardiograma; ETE: ecocardiografia transesofágica; ETT: ecocardiograma transtorácico; FA: fibrilação atrial; ICVP: isolamento circunferencial das veias pulmonares; ICVP plus: inclui ICVP com uma ou mais ablações adjuvantes; NOS: Escala de Newcastle-Ottawa; NR: não relatado; RM-RTG: ressonância magnética com realce tardio de gadolínio; TCC: tomografia computadorizada cardíaca; TCMD: tomografia computadorizada com múltiplos detectores; VAAE: volume do apêndice atrial esquerdo.*

### Comparações de VAAE em pacientes com e sem recorrência de FA após ablação por cateter

**Figura 2:**
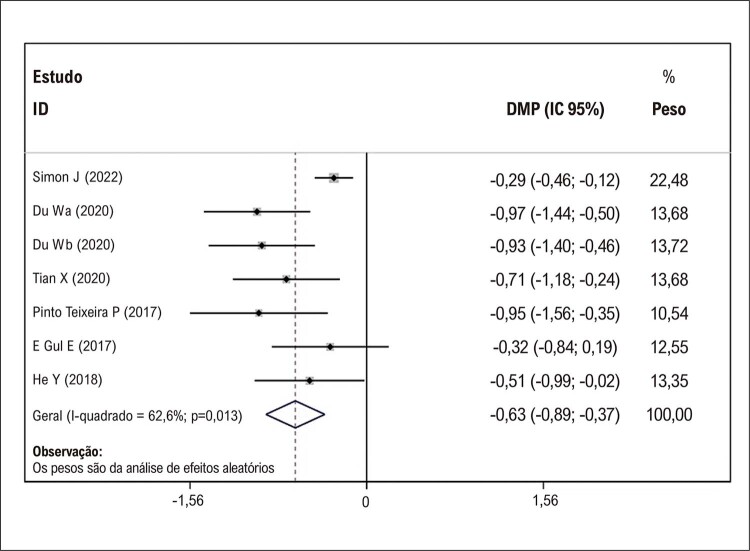
– Gráfico de floresta para as diferenças no volume do apêndice atrial esquerdo basal em pacientes com e sem recorrência de fibrilação atrial. DMP: diferença média padronizada; IC: intervalo de confiança.

**Figura 3:**
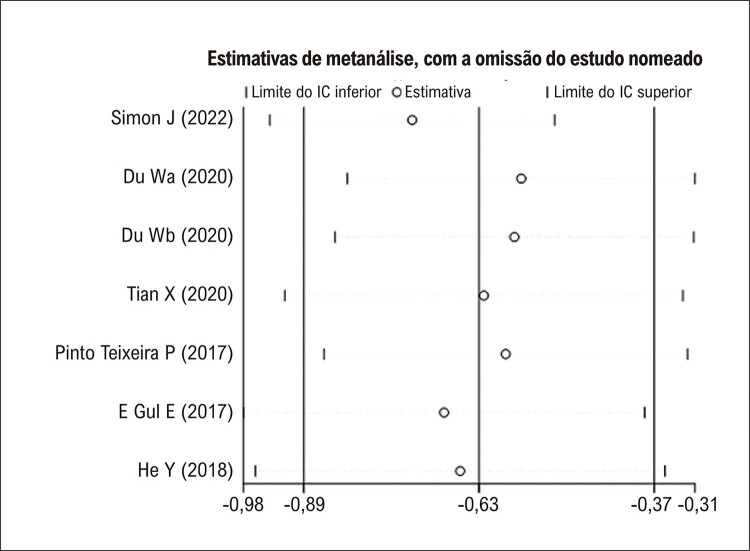
– Análise de sensibilidade da diferença média padronizada do volume do apêndice atrial esquerdo basal em pacientes com e sem recorrência de fibrilação atrial. IC: intervalo de confiança.

**Figura 4:**
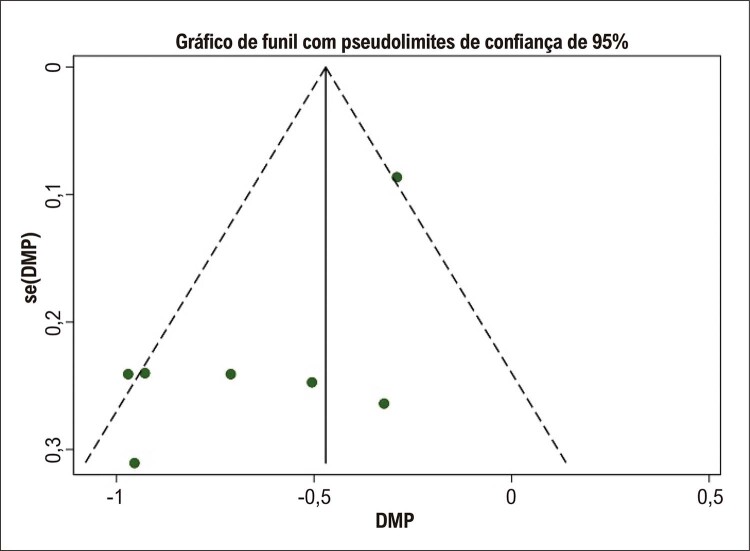
– Gráfico de funil para as diferenças no volume do apêndice atrial esquerdo basal em pacientes com e sem recorrência de fibrilação atrial. DMP: diferença média padronizada; se: erro padrão.

Todos os 6 estudos de coorte incluídos relataram VAAE basal em pacientes que desenvolveram ou não recorrência de FA após ablação por cateter. Du et al. ^8^ utilizaram a média do VAAE derivado da tomografia computadorizada, que foi maior que a média do VAAE medido pela ecocardiografia transesofágica, mas há forte correlação entre elas. Neste caso, incluímos o VAAE médio por meio de ecocardiografia transesofágica ou tomografia computadorizada. Nossa metanálise mostrou que os pacientes com recorrência de FA tiveram uma média maior de VAAE em comparação com pacientes sem recorrência (DMP: −0,63; IC de 95%: −0,89 a −0,37; todos os valores de p < 0,05; Figura 2 ). Na análise de sensibilidade, ao remover um estudo individual um de cada vez, nenhum dos estudos alterou materialmente os resultados resumidos ( Figura 3 ). O gráfico de funil na Figura 4 revelou alguma assimetria na inspeção visual, sugerindo um possível viés de publicação. Esses resultados sugerem que os pacientes que desenvolveram recorrência de FA após ablação por cateter apresentaram maior VAAE pré-procedimento em comparação com aqueles que não desenvolveram recorrência de FA.

### Eficácia preditiva do VAAE basal para determinar o risco de recorrência de FA após ablação por cateter

**Figura 5:**
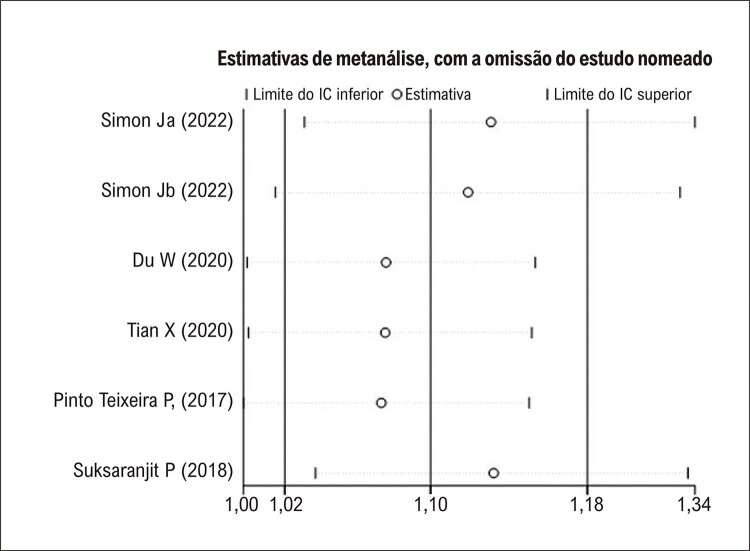
– Análise de sensibilidade dos coeficientes agrupados de hazard ratio na relação entre volume do apêndice atrial esquerdo e risco de fibrilação atrial. IC: intervalo de confiança.

**Figura 6:**
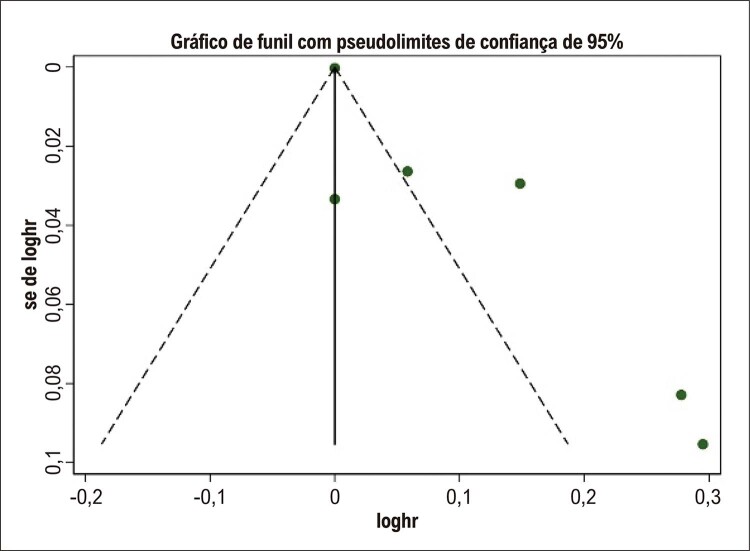
– Gráfico de funil para a eficácia preditiva do volume do apêndice atrial esquerdo basal para o risco de recorrência de fibrilação atrial após ablação por cateter. hr: hazard ratio; se: erro padrão.

Cinco estudos com 878 pacientes relataram a associação multivariada ajustada entre o VAAE basal e o risco de recorrência de FA após ablação por cateter. Simon et al. ^14^ examinaram as diferenças dos parâmetros de imagem entre pacientes com FA paroxística e persistente, e incluímos VAAE em ambos os grupos. Esta metanálise mostrou que o VAAE está associado a uma maior recorrência de FA após ablação por cateter de radiofrequência (HR = 1,10; IC de 95%: 1,02 a 1,18; p = 0,000), conforme mostrado na Figura Central . As análises de sensibilidade, realizadas pela omissão de um estudo de cada vez, obtiveram resultados semelhantes ( Figura 5 ). O gráfico de funil demonstrou assimetria sugerindo possível viés de publicação ( Figura 6 ). Esses resultados sugerem que maior VAAE pode ser um preditor independente de recorrência de FA em pacientes submetidos a ablação por cateter.

**Figura Central:**
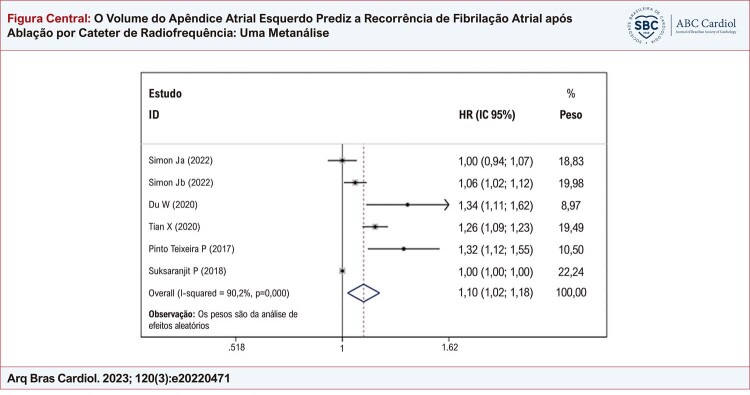
: O Volume do Apêndice Atrial Esquerdo Prediz a Recorrência de Fibrilação Atrial após Ablação por Cateter de Radiofrequência: Uma Metanálise

HR: hazard ratio; IC: intervalo de confiança; ID: identificação.

## Discussão

A ablação por cateter para FA tornou-se uma importante opção de tratamento, e o volume de procedimentos aumentou em todo o mundo desde o seu início em 2000. ^22^ Em um ensaio clínico recente, a ablação por cateter foi associada a reduções no risco composto de morte, acidente vascular cerebral incapacitante, sangramento grave e parada cardíaca. ^23^ No entanto, apesar da rápida evolução das técnicas de ablação de FA, o procedimento apresenta um risco relevante de complicações maiores, especialmente com uma alta taxa de recorrência de FA. Dessa maneira, há a necessidade da previsão preliminar da eficácia da ablação de FA para orientar a seleção de pacientes apropriados e aumentar a relação de benefício dessa estratégia invasiva. Provou-se que o aumento do tamanho do átrio esquerdo é um preditor independente da recorrência de FA. No entanto, a acurácia do volume do átrio esquerdo em prever a recorrência de FA em pacientes pode ser reduzida, devido ao fato de que a morfologia do átrio esquerdo influencia muitos estados patológicos e depende da proficiência do operador. Assim, a reconstrução estrutural do átrio esquerdo pode ser um resultado combinado de múltiplos fatores. Du et al. ^8^ demonstraram que o VAAE tem uma boa correlação com o diâmetro do átrio esquerdo, o volume do átrio esquerdo e o nível de NT-proBNP, sugerindo que o remodelamento do AAE demonstrado pelo VAAE pode ser considerado como parte do remodelamento do átrio esquerdo e pode ser usado para avaliar o desfecho de pacientes com FA após ablação por cateter. O VAAE pode potencialmente fornecer uma avaliação de risco mais precisa em comparação com o tamanho do átrio esquerdo. Os principais achados desta metanálise são os seguintes: (a) pacientes com recorrência de FA apresentaram maior média de VAAE em comparação com pacientes sem recorrência; (b) maior VAAE pode ser um fator de risco para recorrência de FA após ablação por cateter.

O AAE é um remanescente do átrio esquerdo embrionário, enquanto o átrio esquerdo remanescente é derivado de um crescimento das veias pulmonares. ^24^ O AAE é um órgão funcional e estruturalmente complexo que contribui para as alterações hemodinâmicas cardíacas e a frequência cardíaca por meio de suas propriedades contráteis e secreção de peptídeos neuro-hormonais. ^25^ Por um lado, estudos prévios mostraram que o AAE é a fonte mais prevalente de eventos cardioembólicos e está tipicamente associado a arritmias atriais, como FA e flutter atrial. ^26^ Portanto, a avaliação pré-procedimento do átrio esquerdo e do AAE por ecocardiografia transesofágica é convencionalmente realizada para detectar a formação de trombos antes da cardioversão e isolamento das veias pulmonares. Por outro lado, o AAE também demonstrou ser uma fonte de iniciação e manutenção da FA, particularmente em pacientes que necessitam de ablação repetida para recorrências de arritmia. Alguns estudos verificaram que o AAE desencadeia arritmias em até 30% de seus pacientes; assim, eles rotineiramente isolam o AAE no momento da ablação repetida. ^27 , 28^

Poucos estudos anteriores focaram no valor do AAE em relação à recorrência de FA após ablação por radiofrequência. Kanda et al. ^29^ usaram os parâmetros morfológicos e funcionais do AAE como um fator substituto da função atrial esquerda e foram os primeiros a demonstrar que uma baixa velocidade de pico de fluxo do AAE está associada à recorrência de FA após ablação por cateter. No entanto, outro estudo investigou as habilidades da onda P antes do procedimento para o pico da onda A na imagem de Doppler tecidual, o índice de volume do átrio esquerdo e os valores de velocidade de fluxo do AAE para prever a recorrência de FA após ablação por cateter de radiofrequência para FA paroxística, e concluiu a avaliação do remodelamento funcional de FA pela velocidade de fluxo do AAE. ^30^ Porém, em um estudo mais recente, Kocyigit et al. identificaram uma relação entre a morfologia do AAE do tipo “couve-flor” e recorrências após ablação por cateter. ^31^

Posteriormente, alguns estudos de pequena escala também reconheceram que VAAE mais elevado está independentemente associado ao aumento da incidência de recorrência de FA após ablação por cateter em pacientes com FA. Embora os mecanismos potenciais subjacentes à associação entre o AAE e a FA permaneçam obscuros, um grande corpo de evidências indica que o VAAE elevado contribui para o ciclo vicioso de remodelamento atrial e FA. Além disso, a liberação do peptídeo natriurético atrial é desencadeada por receptores de estiramento, sendo a distensão da parede do AAE mais preditiva da liberação do peptídeo natriurético atrial do que a distensão atrial esquerda ou a pressão atrial esquerda. ^32^ Esse peptídeo atua nos receptores do peptídeo natriurético atrial, exercendo assim a sequência de efeitos fisiológicos, incluindo aumento da excreção renal de sódio, redução do volume extracelular, vasodilatação e redução da pressão arterial. Esses fatores podem estar associados ao processo de remodelamento atrial. Portanto, o VAAE pode ser um parâmetro confiável para determinar as condições estruturais e funcionais do átrio esquerdo em pacientes com FA precoce.

Até onde sabemos, nosso estudo é a primeira metanálise a avaliar a potencial associação entre o VAAE e a recorrência de FA após ablação por cateter. É importante compreender o volume e a função do AAE para alcançar um melhor tratamento personalizado em um futuro próximo.

### Limitações

O presente estudo tem várias limitações. Em primeiro lugar, nossa análise incluiu um número limitado de estudos; todos os estudos avaliados foram retrospectivos e nenhum estudo populacional foi realizado na América do Sul. Em segundo lugar, o gráfico de funil revelou alguma assimetria na inspeção visual para nossas duas metanálises, sugerindo possível viés de publicação ( Figura 4 ). O teste de regressão de Egger não foi utilizado devido ao número limitado de estudos incluídos, mas o método “ *trim-and-fill* ” também não obteve a simetria do gráfico de funil. Esses resultados sugerem que nossa metanálise pode ser afetada por viés de publicação. Devido ao viés de publicação, mais estudos devem ser realizados para explorar os mecanismos subjacentes à recorrência da FA. Em terceiro lugar, não estudamos a diferença do VAAE entre FA paroxística e persistente. Em quarto lugar, as modalidades de imagem para avaliar o VAAE variaram consideravelmente entre os estudos incluídos e a precisão dos diferentes métodos de medição tem alguma influência em nossa metanálise. Em quinto lugar, não avaliamos a influência dos parâmetros morfológicos e funcionais do VAAE na geração da arritmia em todos os pacientes no presente estudo. É necessário investigar a possibilidade de outros locais que desencadeiam a FA em pacientes com recorrência de FA e apêndice atrial esquerdo maior como próximo passo.

## Conclusão

Em resumo, nossa metanálise identificou que pacientes com recorrência de FA após ablação por cateter de radiofrequência apresentam VAAE significativamente maiores em comparação com pacientes sem recorrência. O VAAE é relevante para aumentar o risco de recorrência de FA após ablação por cateter de radiofrequência. Entretanto, a avaliação do VAAE nesses pacientes na prática clínica de rotina é importante para melhor estratificação de risco e orientação quanto à melhor opção terapêutica.
