# Supplementary material for: O Volume do Apêndice Atrial Esquerdo Prediz a Recorrência de Fibrilação Atrial após Ablação por Cateter de Radiofrequência: Uma Metanálise
Source: Arq Bras Cardiol. 2023 Mar 7;120(3):e20220471. [Article in Portuguese] doi: 10.36660/abc.20220471 (PMC12080705; doi:10.36660/abc.20220471)
Supplement: Translation

# Translation: Left Atrial Appendage Volume Predicts Atrial Fibrillation Recurrence after Radiofrequency Catheter Ablation: A Meta-Analysis

**Authors:** Zhenghao Liu, Xiaofei Mei, Hezi Jiang, Yujie Cui, Weiwei Yin, Kuangyi Wang, Tan Chen, Yafeng Zhou

**Affiliations:** 1 China Department of Cardiology, Dushu Lake Hospital Affiliated to Soochow University, Medical Center of Soochow University, Suzhou Dushu Lake Hospital, Suzhou – China; 2 China Institution for Hypertension of Soochow University, Suzhou – China

**Keywords:** Heart Failure, Radiofrequency Ablation, Atrial Appendage, Meta-Analysis

## Abstract

**Background:**

The influence of left atrial appendage volume (LAAV) on the recurrence of atrial fibrillation (AF) following radiofrequency catheter ablation remains unclear.

**Objectives:**

We performed a meta-analysis to assess whether LAAV is an independent predictor of AF recurrence following radiofrequency catheter ablation.

**Methods:**

The PubMed and the Cochrane Library databases were searched until March 2022 to identify publications evaluating LAAV in association with AF recurrence after radiofrequency catheter ablation. Seven studies that fulfilled the specified criteria of our analysis were found. We used the Newcastle-Ottawa Scale to evaluate the quality of the studies. The pooled effects were evaluated depending on standardized mean differences (SMDs) or hazard ratios (HRs) with 95% confidence intervals (CIs). P values < 0.05 were considered statistically significant.

**Results:**

A total of 1017 patients from 7 cohort studies with a mean follow-up 16.3 months were included in the meta-analysis. Data from 6 studies (943 subjects) comparing LAAV showed that the baseline LAAV was significantly higher in patients with AF recurrence compared to those without AF (SMD: −0.63; 95% CI: −0.89 to −0,37; all p values < 0.05; I^2^= 62.6%). Moreover, higher LAAV was independently associated with a significantly higher risk of AF recurrence after radiofrequency catheter ablation (HR: 1.10; 95% CI: 1.02 to 1.18).

**Conclusions:**

The meta-analysis showed that there is a significant correlation between LAAV and AF recurrence after radiofrequency catheter ablation, and the role of LAAV in AF patients should not be ignored in clinical practice.

## Introduction

Atrial fibrillation (AF) is the most common cardiac arrhythmia with a worldwide prevalence of around 46.3 million individuals in 2016.^1^AF can lead to stroke, heart failure, dementia, and even death, with a high rate of disability and fatality, thus causing enormous medical and socioeconomic burdens around the world.^2^Catheter ablation is more beneficial than conventional medical therapy in restoring sinus rhythm and long-term quality of life in patients with AF.^3^Pulmonary vein isolation remains the cornerstone of catheter-based treatment for paroxysmal and persistent AF. However, depending on the ablation strategy and the type of AF, the success rates of pulmonary vein isolation after 1 year vary considerably, from 50% to 80%.^4^The overall 1-year success rate of AF ablation, applying the definition of ablation success provided in the 2017 consensus document (freedom from even a single 30-second or longer episode of AF/atrial tachycardia/atrial flutter after the 3-month blanking period off antiarrhythmic drugs), has been observed to be approximately 52%. There are several predictors of AF recurrence following catheter ablation in the literature, such as advanced age; female sex; AF type; genetic predisposition; coexistent comorbidities including obesity, sleep apnea, metabolic syndrome, hypertension, heart failure, and valvular heart disease; and degree of left atrial dilatation and scarring.^5^

The importance of studying the left atrial appendage (LAA) has been growing exponentially, since it has played a vital role in AF. There is a close relationship between the level of LAA flow velocity and the frequency of thrombus and spontaneous echo contrast as qualitative parameters of an elevated thromboembolic risk.^6^Furthermore, according to the BELIEF Trial, LAA isolation outside the pulmonary veins improved prognosis in patients with longstanding persistent AF.^7^Recent studies have confirmed that left atrial appendage volume (LAAV) is involved in the recurrence of AF.^8 - 14^However, these results are conflicted, with some papers reporting that LAAV has a weak correlation or is even irrelevant to patients with AF.^15 , 16^

Therefore, results of previous studies have not been quantitatively summarized in a meta-analysis. We conducted a meta-analysis of these studies to clarify whether baseline LAAV was predictive for AF recurrence after catheter ablation.

## Methods

We conducted this meta-analysis in accordance with the Meta-analysis of Observational Studies in Epidemiology (MOOSE)^17^and the Preferred Reporting Items for Systematic Reviews and Meta-analyses (PRISMA).^18^Since our meta-analysis was based on previously published studies, ethical approval and patient consent were not required.

### Search strategies

We searched the PubMed and Embase databases using the terms “left atrial appendage” , ”ablation” , ”recurrence ”, and “atrial fibrillation”. The search was limited to studies in humans published in English. We also manually searched the reference lists of the related original and review articles for possible studies. The final literature search was performed on March 1, 2022.

### Study selection

The aim of our study was to evaluate the association between baseline LAAV and AF recurrence after catheter ablation. Therefore, we included retrospective reporting either of the following outcomes: (1) mean differences of LAAV between patients with or without AF recurrence after catheter ablation or (2) multivariable adjusted relative risks of AF recurrence after catheter ablation based on per unit increase of baseline LAAV. Studies were required to have a minimum follow-up of 6 months after catheter ablation. For these studies, LAAV was assessed by one or multiple of the following modalities: transthoracic echocardiogram, transesophageal echocardiograghy, cardiac computed tomography, or magnetic resonance imaging.

### Data extraction and quality evaluation

Two authors (L and M) independently performed the literature search, data extraction, and quality assessment according to the predefined inclusion criteria. Discrepancies were resolved by consensus. The extracted data included patient characteristics, numbers of patients with AF included, retrospective or prospective observational studies, mean ages, sex, type of AF, and proportions of patients with coronary artery disease, details of catheter ablation procedures, follow-up durations, and strategies for detecting AF recurrence. For the outcome data, we included studies that fulfilled all the previous criteria and included standardized mean differences (SMDs) of baseline LAAV in patients with and without AF recurrence and hazard ratio (HR) and 95% confidence intervals (CI) of LAAV as predictors of AF recurrence. The quality of the included studies was evaluated using the Newcastle-Ottawa Scale,^19^which judges the quality of each cohort study with regard to 3 aspects: selection of the study groups, comparability of the groups, and ascertainment of the outcome of interest.

### Statistical analyses

For the analysis of mean LAAV in patients with AF recurrence, mean LAAV values were extracted for patients with AF recurrence and patients without AF recurrence, and SMDs and 95% CIs were calculated for each study. To analyze the risk of AF recurrence after radiofrequency catheter ablation based on LAAV, we used standardized HRs with 95% CIs to evaluate differences in LAAV between patients with or without AF recurrence for the meta-analyses. For studies reporting odds ratio (OR) only, OR values using the univariate and multivariate Cox proportional odds model in each primary study were directly considered as HRs. P values < 0.05 were considered to be statistically significant. Cochran’s Q test and I^2^test were performed to evaluate the heterogeneity among studies. I^2^> 50% indicated significant heterogeneity. A random effect or fixed-effect model was used depending on the heterogeneity calculated. For the mean LAAV in AF recurrence analysis, Q statistic (p = 0.013) and I^2^index of 62.6 indicated significant heterogeneity. For analysis of the studies reporting the risk of AF recurrence based on LAAV, Q statistic (p = 0.00) and I^2^index of 90.2 indicated severe heterogeneity again prompting us to adopt the random effect model to pool effect sizes. A random effect model was applied to synthesize the results, because this is a more generalized method that incorporates the heterogeneity of the included studies when combining the results. Sensitivity analyses, conducted by removing individual studies one at a time, were performed to evaluate the stability of the results.^20^Funnel plots and Egger’s regression tests were performed to assess the potential publication bias.^21^All statistical tests were performed with STATA version 15.0 (StataCrop, College Station, TX, USA).

## Results

### Results of literature search

Figure 1 displays the process of the database search and study. Briefly, 170 studies were obtained via our initial literature search. After the removal of duplicates, 132 articles were screened by title and abstract, and 115 of them were excluded (7 studies were not relevant;44 were not cohort studies; 64 were review articles, letters, or editorials). The remaining 17 studies underwent full-text review. Of these, 10 studies were further excluded for the following reasons: not relevant to AF recurrence (n = 2), insufficient data (n = 6), study type (n = 1), and duplicate cohorts of the included studies (n = 1). Ultimately, 7 studies met the criteria and were included in our analysis.

**Figure 1:** – The flow chart of literature identification.

### Study characteristics and quality evaluation

The characteristics of the included studies are listed in Table 1 . Overall, our meta-analysis included 7 retrospective cohort studies with a total of 1017 patients with AF who underwent catheter ablation. Mean follow-up duration of the studies was 16.3 months. One study included paroxysmal AF patients exclusively,^11^and another included persistent AF patients only,^12^while the others included both subtypes of AF. Some studies evaluate LAAV using one or multiple methods including: transthoracic echocardiograghy, transesophageal echocardiograghy, computed tomography, and magnetic resonance imaging. Six of the included studies performed pulmonary vein isolation alone, while one study performed additional linear ablation during AF ablation. The included studies were generally of good study quality, with the Newcastle-Ottawa Scale varying between 6 and 9.

**Table 1:** – Characteristics of the included studies

Study, year	Region	Number of patients	Study designer	Male, %	Age, Years	Type of AF (paroxysmal AF, %)	Follow-up time, months	Hypertension, %	Diabetes, %	CAD, %	Mean LAAV		Imaging used	RFA details	Banking periods, months	Assessment to detect recurrence	NOS
											Recurrence	No recurrence					
Simon J, 2022.^14^	Europe	561	Single centre, retrospective study	65.1	61.9 ± 10.2	40.8	NR	73.3	14.6	9.1	8.8 ± 5.2	7.6 ± 3.2	CCT	CPVI	3	ECG or Holter ECG	9
Du W, 2020.^8^	Asia	108	Single centre, retrospective study	53.7	63.1 ± 8.1	65.7	12	NR	17.6	18.5	13.34	9.67	CCT, TTE, TEE	CPVI plus	3	ECG or Holter ECG	7
Tian X, 2020.^9^	Asia	83	Single centre, retrospective study	59	60.4 ± 10.1	65.7	19	NR	NR	NR	8.53	15.8	CCT	CPVI	3	Holter	9
Teixeira P, 2017.^10^	Europe	52	Single centre, retrospective study	58	54.4 ± 9.7	57.7	24	NR	NR	NR	11.3	8.2	CCT	CPVI	3	ECG or Holter ECG	7
Gul E, 2017.^11^	North America	59	Single centre, retrospective study	44	64.6 ± 9.8	0	13	69	20	19	11	9.7	MDCT, TTE	CPVI	3	Holter	8
He Y, 2018.^12^	Asia	80	Single centre, retrospective study	60	57.3 ± 10.4	100	12	NR	NR	NR	13.3	11.2	TTE, TEE	CPVI	3	ECG or Holter ECG	8
Suksaranjit P, 2018.^13^	North America	74	Single centre, retrospective study	68	72 ± 11	40	18	65	7	11	NR	NR	LGE-MRI	CPVI	3	Holter	7

*All studies adopted a significance level of p < 0.05. AF: atrial fibrillation; CAD: coronary artery disease; CPVI: circumferential pulmonary vein isolation; CPVI plus: includes CPVI with one of more adjuvant ablations; CCT: cardiac computer tomography; ECG: electrocardiogram; LAAV: left atrial appendage volume; LGE-MRI: late gadolinium enhancement magnetic resonance imaging; MDCT: multidetector computed tomography; NR: not reported; NOS: Newcastle-Ottawa Scale; RFA: radiofrequency catheter ablation; TEE: transesophageal echocardiograghy; TTE: transthoracic echocardiogram.*

### Comparisons of LAAV in Patients with and without AF Recurrence after Catheter Ablation

All of the included 6 cohort studies reported baseline LAAV in patients who developed or did not develop AF recurrence after catheter ablation. Du et al.^8^used mean LAAV derived from computed tomography, which was higher than the mean LAAV measured by transesophageal echocardiograghy, but there is a strong correlation between them. In this case, we included mean LAAV using transesophageal echocardiograghy or computed tomography. Our meta-analysis showed that patients with AF recurrence had a higher mean LAAV compared to patients with no recurrence (SMD: −0.63; 95% CI: −0.89 to −0.37; all p values < 0.05; Figure 2 ). In the sensitivity analysis, by removing individual study one at a time, none of the studies changed the summary results materially ( Figure 3 ). The funnel plot in Figure 4 revealed some asymmetry from visual inspection, suggesting potential publication bias. These results suggest that patients who developed AF recurrence after catheter ablation had higher preprocedural LAAV compared to those who did not develop AF recurrence.

**Figure 2:** – Forest plots for the differences in baseline LAAV in patients with and without AF recurrence.

**Figure 3:** – Sensitivity analysis of SMD of baseline LAAV in patients with and without AF recurrence.

**Figure 4:** – Funnel plots for the differences in baseline LAAV in patients with and without AF recurrence.

### Predictive efficacy of baseline LAAV for determining the risk of AF recurrence after catheter ablation

Five studies with 878 patients reported the multivariable adjusted association between baseline LAAV and the risk of AF recurrence after catheter ablation. Simon et al.^14^examined the differences of the imaging parameters between patients with paroxysmal and persistent AF, and we included LAAV in both groups. This meta-analysis showed that LAAV is associated with a higher AF recurrence after radiofrequency catheter ablation (HR = 1.10; 95% CI: 1.02 to 1.18; p = 0.000), as shown in Central Illustration . Sensitivity analyses, conducted by omitting one study at a time, retrieved similar results ( Figure 5 ). The funnel plot demonstrated asymmetry suggesting possible publication bias ( Figure 6 ). These results suggest that higher LAAV may be an independent predictor of AF recurrence in patients undergoing catheter ablation.

**Central Illustration:** : Left Atrial Appendage Volume Predicts Atrial Fibrillation Recurrence after Radiofrequency Catheter Ablation: A Meta-Analysis

**Figure 5:** – Sensitivity analysis of the pooled HR coefficients on the relationship between LAAV and AF risk.

**Figure 6:** – Funnel plots for the predictive efficacy of baseline LAAV for risk of AF recurrence after catheter ablation.

## Discussion

Catheter ablation for AF has become an important treatment option, and the volume of procedures has increased worldwide since its inception in 2000.^22^In a recent clinical trial, catheter ablation was associated with reductions in the composite risk of death, disabling stroke, serious bleeding, and cardiac arrest.^23^However, despite the rapid evolution of AF ablation techniques, the procedure has a relevant risk of major complications, especially with a high AF recurrence rate. Accordingly, there is a need for preliminary prediction of AF ablation effectiveness to guide selection of appropriate patients and increase the benefit ratio of this invasive strategy. Enlarged left atrial size has been proved to be an independent predictor for AF recurrence. However, the accuracy of left atrial volume in predicting AF recurrence in patients may be reduced, due both to the fact that left atrial morphology influences many pathological states, and it depends on the operator’s proficiency. Thus, the structural reconstruction of the left atrium may be a combined result of multiple factors. Grzegorz Kiełbasa et al.^8^demonstrated that LAAV has a good correlation with left atrial diameter, left atrial volume and NT-proBNP level, suggesting that LAA remodeling demonstrated by LAAV could be considered as part of left atrial remodeling and could be used to evaluate the outcome of patients with AF after catheter ablation. LAAV can potentially provide a more accurate risk assessment compare to left atrial size. The key findings of this meta-analysis are as follows: (a) Patients with AF recurrence had a higher mean LAAV compared to patients with no recurrence; (b) higher LAAV may be a risk factor for AF recurrence after catheter ablation.

The LAA is a remnant of the embryonic left atrium, while the remaining left atrium is derived from an outgrowth of the pulmonary veins.^24^The LAA is a structurally complex and functional organ that contributes to cardiac hemodynamic changes and heart rate through both its contractile properties and neurohormonal peptide secretion.^25^On the one hand, prior studies have shown that the LAA is the most prevalent source of cardioembolic events, and it is typically associated with atrial arrhythmias such as AF and atrial flutter.^26^Therefore, preprocedural evaluation of the left atrium and LAA by transesophageal echocardiograghy is conventionally performed to detect thrombus formation prior to cardioversion and pulmonary vein isolation. On the other hand, the LAA has also been shown to be a source of initiation and maintenance of AF, particularly in patients requiring repeat ablation for arrhythmia recurrences. Some studies have found that the LAA triggers in up to 30% of their patients; thus, they are routinely isolating the LAA at the time of repeat ablation.^27 , 28^

Few studies have focused on the value of the LAA in AF recurrence following radiofrequency ablation in the past. Kanda et al.^29^used the morphological and functional parameters of the LAA as a surrogate factor of left atrial function, and they were the first to demonstrate that a low LAA peak flow velocity is associated with AF recurrence after catheter ablation. Meanwhile, another study investigated the abilities of the preprocedural P-wave to the peak A-wave on the tissue Doppler imaging, left atrial volume index, and LAA flow velocity values to predict AF recurrence after radiofrequency catheter ablation for paroxysmal AF, and concluded evaluation of functional remodeling of AF by LAA flow velocity.^30^However, in a more recent study, Kocyigit et al. identified a relationship between cauliflower-type LAA morphology and recurrences after catheter ablation.^31^

Subsequently, some small-scale studies also recognized that higher LAAV is independently associated with an increased incidence of AF recurrence after catheter ablation in patients with AF. Although potential mechanisms underlying the association between LAA and AF remain unclear, a large body of evidence indicates that high LAAV contributes to the vicious cycle of atrial remodeling and AF. In addition, atrial natriuretic peptide release is triggered by stretch receptors, with LAA wall distention being more predictive of atrial natriuretic peptide release than left atrial distention or left atrial pressure.^32^This peptide acts on atrial natriuretic peptide receptors, thereby exerting the sequence of physiological effects, including increased renal sodium excretion, reduced extracellular volume, vasodilation, and reduced blood pressure. These factors may be associated with the process of atrial remodeling. Therefore, LAAV could be a reliable parameter for determining the left atrial structural and functional conditions in patients with early AF.

To our knowledge, our study is the first meta-analysis to evaluate the potential association between LAAV and AF recurrence after catheter ablation. It is important to understand LAAV and function for better personalized treatment in the near future.

### Limitations

The present study has several limitations. Firstly, our analysis included a limited number of studies;all evaluated studies were retrospective, and no populational study was conducted in South America. Secondly, the funnel plot revealed some asymmetry upon visual inspection for our two meta-analyses, suggesting possible publication bias ( Figure 4 ). The Egger’s regression test was not used due to the limited number of included studies, but “trim-and-fill” method also did not achieve symmetry of the funnel plot. These results suggest that our meta-analysis may be affected by publication bias. Because of publication bias, further studies should be performed to explore the mechanisms underlying AF recurrence. Thirdly, we did not study the difference of the LAAV between paroxysmal and persistent AF. Fourthly, the imaging modalities to assess LAAV varied considerably in the included studies, and the precision of different measure methods has some influence on our meta-analysis. Fifthly, we did not evaluate the influence of morphological and functional parameters of LAAV on arrhythmia generation in all patients in the present study. It is necessary to investigate the possibility of other AF trigger sites in patients with AF recurrence and larger LAA as a next step.

## Conclusion

In summary, our meta-analysis identified that patients with AF recurrence after radiofrequency catheter ablation have significantly higher LAAV compared to patients without recurrence. LAAV is relevant to increased risk of AF recurrence after radiofrequency catheter ablation. Meanwhile, the assessment of LAAV in these patients in routine clinical practice is important for better risk stratification and guidance regarding the optimum therapeutic option.
